# The Antidepressant-like Activity and Cognitive Enhancing Effects of the Combined Administration of (*R*)-Ketamine and LY341495 in the CUMS Model of Depression in Mice Are Related to the Modulation of Excitatory Synaptic Transmission and LTP in the PFC

**DOI:** 10.3390/ph16020288

**Published:** 2023-02-14

**Authors:** Agnieszka Pałucha-Poniewiera, Bartosz Bobula, Anna Rafało-Ulińska

**Affiliations:** 1 Department of Neurobiology, Maj Institute of Pharmacology, Polish Academy of Sciences, Smętna Street 12, 31-343 Krakow, Poland; 2 Department of Physiology, Maj Institute of Pharmacology, Polish Academy of Sciences, Smętna Street 12, 31-343 Krakow, Poland

**Keywords:** antidepressant, CUMS, LTP, LY341495, mGlu_2_ receptor, (*R*)-ketamine

## Abstract

(*S*)-Ketamine is the first rapid-acting antidepressant drug (RAAD) introduced for the treatment of depression. However, research is still being carried out on the search for further RAADs that will be not only effective but also safe to use. Recent data have indicated that the combined administration of (*R*)-ketamine and the mGlu_2/3_ receptor antagonist LY341495 (mixRL) induces rapid and sustained effects in the chronic unpredictable mild stress (CUMS) model of depression in mice, and the use of this drug combination is associated with a low risk of undesirable effects. Considering the possible influence of stress on cortical plasticity and, on the other hand, the role of this plasticity in the mechanism of action of ketamine, we decided to investigate whether mixed RL affects synaptic plasticity in the prefrontal cortex (PFC) in the CUMS model of depression using electrophysiological techniques and explore whether these effects are related to memory impairments. Using behavioral methods, we found that a single administration of mixRL reversed CUMS-induced PFC-dependent memory deficits and alleviated depression-like effects induced by CUMS. In turn, electrophysiological experiments indicated that the amplitude of field potentials as well as paired-pulse responses in CUMS mice were increased, and mixRL was found to reverse these effects. Additionally, the magnitude of long-term potentiation (LTP) was reduced in CUMS mice, and mixRL was shown to restore this parameter. In summary, mixRL appeared to exert its antidepressant effects and cognitive enhancing effects in a mouse model of depression, at least in part, by mechanisms involving modulation of glutamatergic transmission and LTP in the PFC.

## 1. Introduction

Despite the wide availability of antidepressant drugs (ADs), depression is still a growing clinical and social problem. Both epidemiological and clinical data show that approximately 30% of major depression disorder (MDD) patients suffer from treatment-resistant depression (TRD) [1], and several weeks of waiting for a therapeutic effect is one of the major factors that inhibits the effective therapy of depression. The proposed solution to these problems could be a RAAD, on which research has been conducted for over 20 years, based on an initial observation that (*R,S*)-ketamine may rapidly reduce the symptoms of depression in MDD patients [2]. As a measurable effect of these studies, one of the N-methyl-d-aspartate (NMDA) receptor antagonists, (*S*)-ketamine, was recently introduced into therapy of TRD as a first RAAD [3]. However, there is a high risk of (*S*)-ketamine-induced undesirable effects, which has prompted the search for new, effective, and safe forms of therapy for this disease.

Several studies indicate that (*R*)-ketamine can meet these expectations because it has much weaker and fewer side effects than (*S*)-ketamine or (*R,S*)-ketamine, and both preclinical studies [4] and pilot probes on patients [5] indicate its potential therapeutic efficacy in the treatment of depression.

On the other hand, research is being conducted on reducing the effective dose of ketamine by co-administering it with other substances with a glutamatergic profile [6]. Recent animal data indicate that mGlu_2_ receptor ligands could be good candidates for this purpose. First, it has been found that the mechanism of (*R,S*)-ketamine’s effect depended on mGlu_2_ receptor action [7], and second, the rapid and prolonged antidepressant effects of (*R,S*)-ketamine were enhanced by the mGlu_2/3_ receptor antagonist LY341495 in an animal model of depression in mice, namely CUMS, as well as in screening tests aimed at looking for antidepressant-like effects in rats, which ultimately allowed for the reduction of the therapeutic dose of ketamine [8,9].

A recent study by Rafało-Ulińska et al. [10] provides new insight into the involvement of specific ketamine enantiomers in this effect. It has been shown that a subeffective dose of only one ketamine enantiomer, (*R*)-ketamine, co-administered with a subeffective dose of LY341495, is able to reverse the stress-induced behavioral effects in the CUMS model of depression. At the same time, subeffective doses of (*S*)-ketamine combined with LY341495 are not effective in this depression model, although (*S*)-ketamine alone, at higher doses, successfully reverses the behavioral effects induced by CUMS. The antidepressant efficacy of (*R*)-ketamine given jointly with a mGlu_2_ antagonist has been confirmed by reversal of the CUMS-induced behavioral effects, including decreased grooming time (reflecting apathy), decreased sucrose preference (indicating anhedonia), and increased immobility in the tail suspension test (TST), and the effectiveness of chosen doses of both substances used in the combination (1 mg/kg of (*R*)-ketamine and 0.3 mg/kg of LY341495) has repeatedly been confirmed in subsequent experiments and labeled mixRL [10]. Importantly, mixRL did not change the locomotor activity of mice in the hyperlocomotion test, in contrast to (*R,S*)-ketamine, which induced substantial hyperlocomotion after a dose typically used as an effective antidepressant dose in animal studies (10 mg/kg), indicating a potential psychostimulatory effect [10].

All these results indicate an advantage of mixRL over the (*S*)-ketamine currently used in the treatment of depression, not only in terms of effectiveness but also in terms of safety. Promising results indicating that mixRL could meet the criteria of a new effective RAAD prompted us to start research on the mechanism responsible for this effect.

A growing body of evidence suggests that the mechanism of the rapid antidepressant-like effects of ketamine, its enantiomers, and some metabolites in rodents are closely related to neuronal plasticity processes in the PFC. Namely, it has been shown that medial PFC (mPFC) pyramidal neurons are disturbed under chronic stress exposure in rodents [11], and ketamine, at doses inducing antidepressant effects, is able to reverse several devastating effects of prolonged stress. Importantly, ketamine increases the density and function of spine synapses in PFC pyramidal neurons [12].

Thus, it was interesting to investigate the role of processes related to PFC functions in the mechanism of action of mixRL. For this purpose, mixRL was used in the CUMS model, and in addition to examining its rapid antidepressant-like effects, electrophysiological studies were performed. Excitatory synaptic transmission, short-term synaptic plasticity (paired-pulse responses), and long-term potentiation (LTP) were analyzed to investigate the influence of mixRL on the modulation of synaptic plasticity in the PFC of mice under CUMS conditions. Since chronic stress may be associated with learning and memory impairments in rodents, we also decided to investigate the possible influence of CUMS and the tested compounds on learning and memory using a behavioral test designed to study memory processes related to PFC activity, namely the temporal order memory task (TOMT).

The studies planned in this way were aimed not only at explaining the mechanism of the antidepressant effect of mixRL in the CUMS model of depression and determining the role of PFC in this process, but they also tried to answer the question whether the combination of drugs used affected learning and memory, which might determine the safety of mixRL as an antidepressant.

## 2. Results

### 2.1. Effect of (R)-Ketamine Co-Administered with LY341495 (mixRL) in the Temporal Order Memory Task in the CUMS Model of Depression in Mice

**Figure 1:**
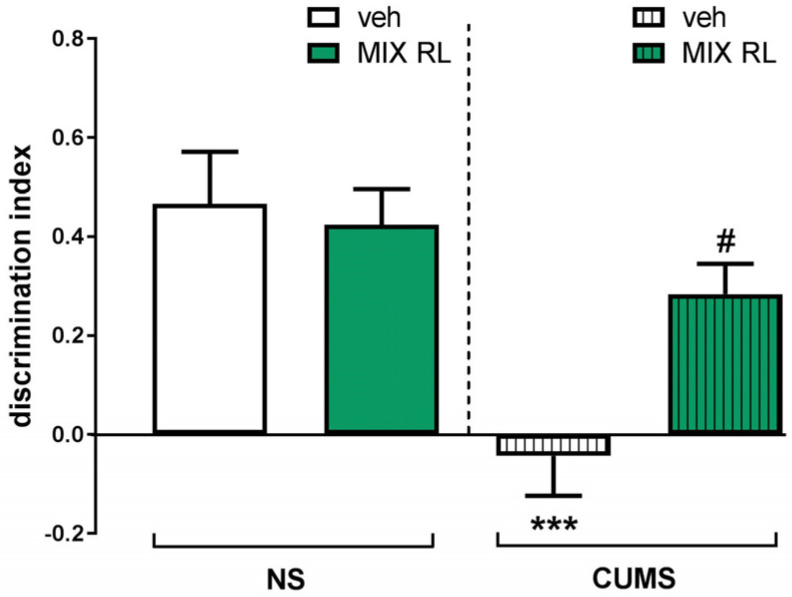
Effect of (*R*)-ketamine (1 mg/kg) co-administered with LY341495 (0.3 mg/kg) (mixRL) on the discrimination index in the temporal order memory task in the CUMS model of depression in mice. The values are expressed as the means ± SEM and were analyzed by two-way ANOVA. *** *p* < 0.001 CUMS main effect; ^#^ *p* < 0.05 CUMS × drug interaction (N = 10). mixRL—*(R)*-ketamine 1 mg/kg + LY 341495 0.3 mg/kg.

CUMS was found to induce a significant disturbance of temporal order memory in TOMT. Two-way ANOVA revealed the main effect of CUMS on the discrimination index between two objects (O1 and O2) (F(1,35) = 16.42, *p* < 0.001). Statistical analysis also showed an interaction between the two parameters (CUMS and mixRL) (F(1,35) = 5.283, *p* < 0.05), which suggests an ability of mixRL to reverse the CUMS-induced memory deficits in TOMT (Figure 1).

### 2.2. Antidepressant-like Effects of (R)-Ketamine Co-Administered with LY341495 (mixRL) in the CUMS Model of Depression in Mice

**Figure 2:**
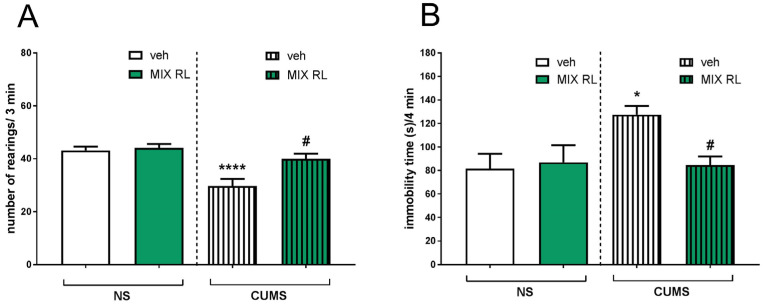
Antidepressant-like effects of (R)-ketamine co-administered with LY341495 (mixRL) in the CUMS model of depression in mice. (**A**) Effect of mixRL on the exploratory behavior in the NCT; (**B**) effect of mixRL on the immobility time in the FST. The values are expressed as the means ± SEM and were analyzed by two-way ANOVA. * *p* < 0.05, **** *p* < 0.0001 CUMS main effect; **^#^** *p* < 0.05 CUMS × drug interaction (N = 8–10). mixRL—(R)-ketamine 1 mg/kg + LY 341495 0.3 mg/kg.

A decreased number of exploratory rearings in CUMS compared to that of NS animals was found in the novel cage test (NCT). Two-way ANOVA showed the main effect of CUMS on this parameter (F(1,35) = 19.48, *p* < 0.0001). Furthermore, an interaction between CUMS and mixRL was found (F(1,35) = 5.509, *p* < 0.05), indicating that mixRL influenced CUMS-induced reduction in exploratory behavior (Figure 2A).

In the forced swim test (FST), a significant increase in the immobility time of the CUMS mice was observed compared to that of the unstressed controls. Two-way ANOVA showed the main CUMS effect (F(1,31) = 4.554, *p* < 0.05) and an interaction between two parameters, CUMS and mixRL (F(1,31) = 5.505, *p* < 0.05), confirming the ability of mixRL to reduce CUMS-induced effects in this test (Figure 2B).

### 2.3. Effects of (R)-Ketamine Co-Administered with LY341495 (mixRL) on the Locomotor Activity of Mice in the CUMS Model of Depression

**Figure 3:**
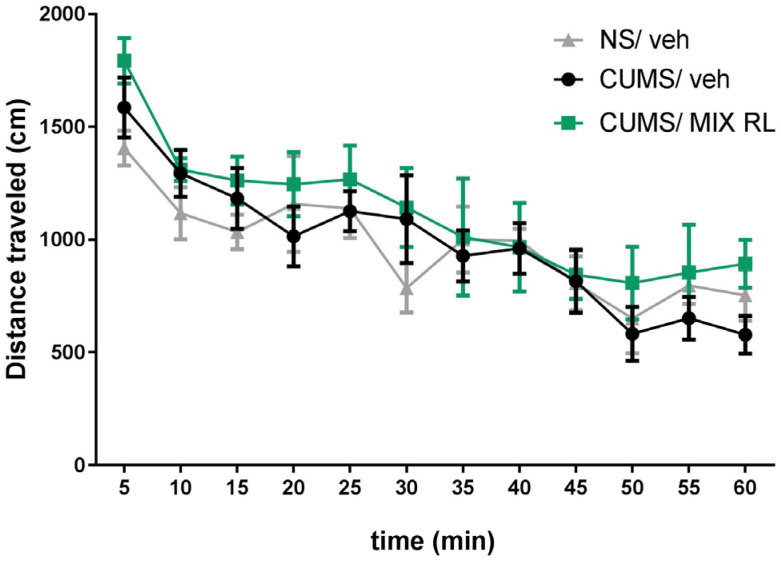
Effect of (R)-ketamine (1 mg/kg) co-administered with LY341495 (0.3 mg/kg) (mixRL) on the locomotor activity of mice. The values are expressed as the means ± SEM and were analyzed by repeated-measures ANOVA (N = 6).

A two-way repeated-measures ANOVA showed a lack of effect of CUMS on the locomotor activity of mice (F(1,10) = 0.02492; *p* > 0.05) compared to that in NS mice. However, a main time effect was found (F(11,110) = 10.11; *p* < 0.0001). Furthermore, the analysis did not reveal an interaction between CUMS and NS (F(11,110) = 0.9478; *p* > 0.05) (Figure 3). A two-way repeated-measures ANOVA also showed that mixRL did not change the locomotor activity of the CUMS mice (F(1,10) = 1.115; *p* > 0.05). At the same time, the main effect of time was revealed (F(11,110) = 12.34; *p* < 0.0001), and there was no interaction between the parameters (CUMS and mixRL) (F(11,110) = 0.3883; *p* > 0.05) (Figure 3).

### 2.4. Effects of (R)-Ketamine Co-Administered with LY341495 (mixRL) in the CUMS Model of Depression on Field Potential (FP) Recording and Paired-Pulse Stimulation

**Figure 4:**
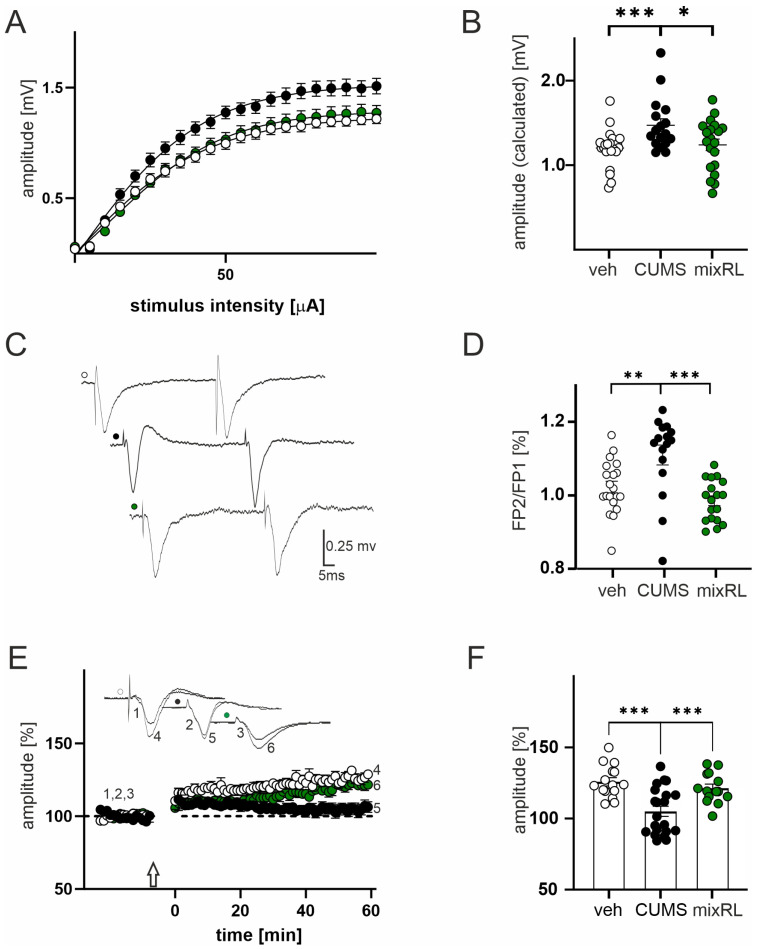
Effect of (R)-ketamine (1 mg/kg) co-administered with LY341495 (0.3 mg/kg) (mixRL) in the CUMS model of depression on field potential (FP) recording, paired-pulse stimulation, and LTP induction. (**A**) Effects of veh, CUMS, and mixRL treatment on the relationship between stimulus intensity and the amplitude of FPs (mean ± SEM) recorded in slices originating from veh animals (white circles, N = 24), stressed animals (CUMS; black circles, N = 17), and mixRL animals (green circles, N = 24); (**B**) differences in maximal FPs amplitudes between veh, CUMS, and mixRL groups; (**C**) raw waveforms of individual FPs evoked by paired stimuli in slices from veh (white circles), after CUMS (black circles) and mixRL (green circles) animals; (**D**) differences in FP2/FP1 between veh, CUMS, and mixRL groups; (**E**) effects of CUMS and mixRL on LTP; plot of the amplitude of FPs (mean ± SEM) recorded in the veh group (white circles, N = 24), stressed group (CUMS; black circles, N = 17), and mixRL (green circles, N = 20); the arrow denotes the time of the beginning of TBS; insets show the superposition of averaged FPs recorded in representative experiments at the times indicated by numbers; (**F**) comparison of the CUMS and mixRL evoked changes in LTP magnitude. The values are expressed as the means ± SEM and were analyzed by one-way ANOVA followed by post hoc Tukey’s multiple comparisons test. * *p* < 0.05, ** *p* < 0.01, *** *p* < 0.001 vs. veh or CUMS, respectively.

One-way ANOVA revealed a significant group effect on field potential amplitude (F(2,58) = 5.922; *p* < 0.01). In slices obtained from mice subjected to CUMS, we observed a significant increase in the maximal evoked FP amplitude in comparison to that in slices obtained from the non-stressed vehicle-treated group (*p* < 0.001, post hoc Tukey’s multiple comparisons test, Figure 4A,B; black and white circles, respectively). 

In slices prepared from stressed mice receiving mixRL, the maximal FP amplitude was comparable to slices obtained from the non-stressed vehicle group (*p* > 0.05, post hoc Tukey’s multiple comparisons test, Figure 4A,B; green circles vs. white circles), but it was significantly lower than in the CUMS group (*p* < 0.05, post hoc Tukey’s multiple comparisons test, Figure 4A,B; green circles vs. black circles). Parameters characterizing the input–output curves of FPs of all experimental groups, calculated using Boltzmann its, are summarized in Table 1.

One-way ANOVA revealed a significant group effect on PPR (F(2,51) = 10.75; *p* < 0.0001). CUMS animals had a significantly higher PPR than non-stressed vehicle-treated animals did (*p* < 0.01, post hoc Tukey’s multiple comparisons test, Figure 4C,D), whereas the mixRL group did not differ from the non-stressed vehicle group (*p* > 0.05, post hoc Tukey’s multiple comparisons test), but it had a significantly lower PPR than the CUMS group did (*p* < 0.001, post hoc Tukey’s multiple comparisons test, Figure 4C,D).

### 2.5. Effects of (R)-Ketamine Co-Administered with LY341495 (mixRL) in the CUMS Model of Depression on LTP Induction

Data analysis revealed a significant group effect on LTP (F(2,47) = 12.08, *p* < 0.0001) with one-way ANOVA. The mean amplitude recorded from non-stressed vehicle-treated animals (measurement between 60 and 65 min after TBS stimulation) was 125.8 ± 2.9% of baseline. LTP was reduced in cortical tissue prepared from CUMS animals (104 ± 3.5%, *p* < 0.001, post hoc Tukey’s multiple comparisons test; Figure 4E,F). In slices prepared from mixRL-treated animals, the LTP increment was not different from that recorded in slices prepared from non-stressed vehicle mice (120.1 ± 2.8%, *p* > 0.05, post hoc Tukey’s multiple comparisons test, Figure 4E,F), but it was significantly lower than that in the CUMS group (*p* < 0.001, post hoc Tukey’s multiple comparisons test; Figure 4E,F).

## 3. Discussion

It was found in this study that the combined administration of (*R*)-ketamine and mGlu_2_ receptor antagonist LY341495, marked as mixRL, not only induced rapid, antidepressant-like effects in a mouse model of depression but also reversed the CUMS-induced memory impairment in the PFC-dependent learning task. Furthermore, using electrophysiological techniques, mixRL was found to prevent the CUMS-induced modifications of glutamatergic transmission and synaptic plasticity in the mouse PFC. CUMS, which is associated with chronic, unpredictable mild stress, is considered to be an effective model of depression that reflects various aspects of the disease, including anhedonia and apathy [13]. It allows not only the study of potential antidepressant effects but also facilitates the identification of the so-called RAADs that work after single or short-term dosing, unlike classic ADs that require long-term administration [8]. The duration of the stress-inducing procedure in C57BL/6J mice was chosen based on previous studies showing that stress-induced depression-related behavioral effects are most pronounced two to three weeks after the start of CUMS, and then they gradually disappear [14]. Generally, after four to five weeks of CUMS, the signs of depression-related behavior are small or negligible, which may indicate that adaptation mechanisms develop in this strain of mice [14]. This depression model has previously been used to reveal high, rapid, and sustained antidepressant-like efficacy of mixRL (1 mg/kg of (*R*)-ketamine and 0.3 mg/kg of LY341495) [10] using the splash test, the sucrose preference test, and the TST. Therefore, the CUMS model was consistently applied in this study to investigate the mechanisms involved in the antidepressant-like action of mixRL. Using subsequent behavioral procedures (FST, NCT), the antidepressant-like effects of a single dose of mixRL were investigated. The results showed that mixRL reduced the CUMS-increased immobility time in the FST 48 h after dosing. Furthermore, based on Strekalova et al. [15], who showed that the reduced exploration of novelty in animals subjected to long-term stress is correlated with anhedonia and not with chronic stress per se, we examined the influence of mixRL on exploratory behavior in the NCT. CUMS significantly reduced exploration behavior in this test, and a single administration of mixRL reversed this effect. This confirms previous results indicating the significant impact of mixRL on the weakening of CUMS-induced anhedonia in mice [10], thus suggesting that mixRL could be an effective and safe RAAD. Additionally, the study of locomotor activity showed no difference in the mobility of the animals given mixRL versus those given the vehicle, indicating the specificity of the antidepressant effect in the FST and the NCT.

Since ketamine and other potential RAADs exert their antidepressant effects by regulating neuroplasticity processes related to the activity of the PFC, we set out to investigate the effect of a potential RAAD, mixRL, on cortical synaptic transmission and cortical synaptic plasticity, with particular emphasis on LTP, which is closely related to regulation of the strength of synaptic transmission and the formation of new synapses in many neural networks, including the PFC.

Here, we found that in slices obtained from CUMS animals, the amplitude of field potentials as well as paired-pulse responses, reflecting short-term synaptic plasticity, were increased, and these effects were prevented by mixRL administration. The increased amplitude of field potentials indicating enhanced glutamatergic transmission in the PFC is consistent with previous findings showing that different kinds of stress enhance glutamate release in the PFC and hippocampus and affect the glutamatergic–GABAergic balance in these structures, which includes the reduction of GABAergic electrophysiological responses and the increase in glutamatergic neurotransmission [16,17,18,19,20]. Importantly, the sustained elevation of extracellular concentrations of glutamate in the PFC and hippocampus likely contributes to the pathogenesis of depression, which in turn became the basis for the search for new antidepressants among substances modulating glutamatergic transmission [21,22]. It has also been found that chronic administration of ADs, including imipramine and citalopram, decreases field potentials in the rat frontal cortex, indicating attenuation of glutamatergic neurotransmission [23]. Furthermore, reduced glutamate release has been shown in rat PFC after repeated treatment with imipramine [24] or citalopram [25]. Thus, it can be assumed that the effect of classical ADs is at least partly due to the attenuation of glutamatergic transmission in the PFC. On the other hand, a RAAD, ketamine, which is known to induce rapid glutamate release in the PFC [26], has been recently shown to induce the opposite effects in stressed and non-stressed rats [27]. In naïve animals, it induces a mild increase in glutamate release in the PFC, but in stressed rats, it inhibits the stress-induced enhancement of glutamate release 24 h and 72 h after a single administration [27], thus indicating that the action of ketamine on glutamatergic transmission may depend on stress conditions.

Using electrophysiological techniques, we also showed that the magnitude of LTP in the PFC was reduced in CUMS animals compared to that in non-stressed subjects, and mixRL significantly reversed this effect. A widely shared hypothesis is that the enhancement of glutamatergic transmission under stress conditions plays a critical role in the modulation of the neuroplasticity processes, including LTP; however, it depends on both the kind and duration of stress [28,29]. Different types of prolonged stress (including crowding stress, restrained stress, or prenatal stress) or chronic corticosterone treatment have been reported to increase cortical synaptic transmission and, at the same time, impair LTP in this brain region [16,30,31]. Furthermore, classic antidepressants, such as imipramine, were shown to reverse some of these effects after repeated administration [30], which suggests an important role of modulation of glutamatergic transmission and LTP in the antidepressant action-related mechanisms. Additionally, in the CUMS model in rats, the influence of chronic stress on the impairment of LTP in the mPFC has been shown [32]. Furthermore, a single dose of rapastinel (GLYX-13), which, like mixRL, is a potential RAAD, reverses these effects and restores LTP efficiency to the control level 24 h after treatment [32]. LTP impairments under CUMS in rats have also been shown in the hippocampus, and (*S*)-ketamine, administered for seven days, alleviates these effects in a Rac1 GTPase-dependent manner [33]. Similarly, when using the Wistar Kyoto (WKY) rat model of depression, LTP impairment was observed in the hippocampus, and a single low dose (5 mg/kg, ip) of ketamine or its metabolite, (2R,6R)-HNK, rescues these LTP deficits at 3.5 h following injection, with residual effects at 24 h [34]. In addition, low-dose ketamine has been shown to be effective in increasing the amplitude of LTP in the hippocampus in mice subjected to chronic social defeat stress (CSDS) [35]. Altogether, it seems that an increase in LTP, especially under conditions of chronic stress modulating depression, correlates with or at least accompanies the antidepressant effects of RAADs. Thus, based on the present study results, it may be supposed that modulation of glutamatergic transmission and LTP in the PFC could be involved in the mechanism of the antidepressant-like effect of mixRL in the CUMS model of depression in mice.

LTP is closely related to different forms of plasticity, including learning and memory processes [36]. Learning and memory disturbances are related to most psychiatric disorders, including depression [37]. They have also been found in animal models of depression involving those based on chronic stress [38]; however, data on the effects of stress on learning and memory are often divergent. On the one hand, research indicates a beneficial effect of stress, especially acute stress, on learning processes, but there are also data indicating learning and memory disturbances in subjects under long-term stress accompanying some diseases, e.g., major depression or neurodegenerative diseases [19].

Therefore, we decided to investigate the effect of CUMS on learning and memory and the influence of mixRL on this parameter. Stress effects on learning and memory and LTP in rats and mice are mainly investigated in the hippocampus, and hippocampus-dependent cognitive deficits, measured as changes in spatial memory, are generally considered to be crucial features of clinical depression. However, both behavioral and electrophysiological evidence suggests that the PFC also participates in memory processes, including recognition memory mechanisms, which are disturbed under chronic stress [19]. It has also been found that distinct recognition memory processes relate to the involvement of different brain regions [39]. The TOMT was found to be specifically related to mPFC activity [39], and we decided to use this behavioral test in our study. The experiment clearly showed that CUMS significantly impaired the discrimination index in vehicle-treated mice. However, mixRL reversed this effect 24 h after treatment, indicating an improvement in object recognition memory in the test used. Thus, it seems that mixRL-induced reversal of CUMS-attenuated LTP may be accompanied by improved recognition memory and that the PFC may be involved in this process. The beneficial effect of another RAAD, ketamine, on LTP and learning and memory has also been observed in studies using another model of chronic stress in mice, namely the CSDS. It was shown that CSDS-induced impairment in hippocampal LTP accompanied by working memory and contextual fear memory deficits is reversed by low-dose ketamine in mice [35]. Furthermore, in the WKY model of depression in rats, where long-term (24 h) memory was impaired, both low-dose ketamine and its active metabolite 2R,6R-HNK have been found to significantly restore recognition memory in the novel object location recognition task, related to hippocampus-dependent spatial memory [34]. Based on the above studies and our results, it seems therefore that ketamine and other RAADs, such as mixRL, can reverse stress-induced memory impairment related not only to hippocampal functions but also to PFC activity.

## 4. Materials and Methods

### 4.1. Animals and Housing

The experiments were performed on male C57BL/6J mice (Charles River, Sulzfeld, Germany) that were 5 weeks of age at the beginning of the experiment. The mice were maintained under standard laboratory conditions in terms of temperature (22 ± 2 °C), humidity (55 ± 10%), and lighting (light phase 6:00–18:00). The mice used in the CUMS model of depression were divided into two groups: control mice (unstressed, designated NS) and mice subjected to CUMS. The animals had constant access to food and water, except for the periods of time in which they were subjected to the action of some stressors that prevented such access (e.g., restraint stress in a plastic tube). Each experimental group consisted of eight to twelve animals.

All experimental procedures were conducted in accordance with the guidelines of the National Institutes of Health Animal Care and Use Committee and were approved by the Second Local Ethics Committee in Kraków, Poland. Every effort was made to reduce the number of animals used and to avoid and minimize animal suffering.

### 4.2. Compounds

(*R*)-Ketamine hydrochloride (Cayman Chemicals, Ann Arbor, MI, USA) and LY341495 disodium salt (Tocris Cookson, Ltd., Bristol, UK) were diluted with 0.9% NaCl. The compounds and vehicles were injected intraperitoneally (i.p.) in a constant volume of 10 mL/kg.

### 4.3. CUMS Procedure

**Figure 5:**
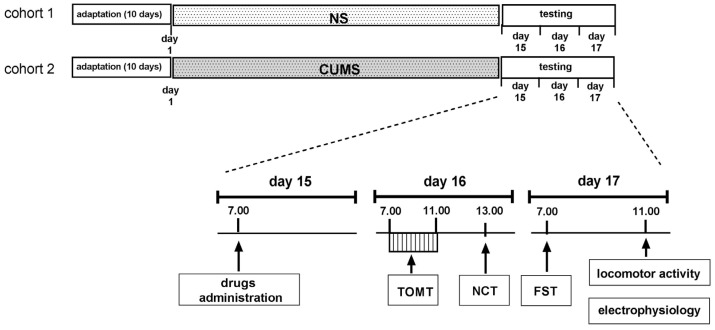
The schedule of the CUMS experiments.

The CUMS procedure was performed as described previously with the necessary modifications [8,39]. It started after 10 days of adaptation to the room conditions, during which all animals were handled and weighed. The following stressors were used: cage tilting (45°; 4–12 h), restraint stress (30–60 min), wet bedding (3–6 h), predator smell (15 min–4 h), removal of sawdust (2–4 h), placing a mouse in a nondomestic cage (1–2 h), reversed light–dark cycle (48 h), substitution of sawdust with 37 °C water (60–90 min), 3 mice in an empty cage (30–60 min), 3 individuals in a cage with 37 °C water (60–90 min), and overcrowding (18 individuals; 60 min). Two or three stressors were used daily, depending on their duration. The stressors started between 7:00 and 11:00 and were administered in random order. To maintain the principle of unpredictability, the duration of stressors was changed. A break of two hours between stressors was used. Some stressors were used at night (reversed day/night cycle, tilted cage) (Figure 5).

On the 15th day after the beginning of CUMS, (*R*)-ketamine (1 mg/kg) was administered jointly with LY341495 (0.3 mg/kg) at 7:00. NaCl (0.9%) was used as a vehicle. Twenty-four hours after the treatment, TOMT (see Section 4.4.1) was performed (starting at 7:00), followed by the NCT (see Section 4.4.2), starting at 13:00. The next day, at 7:00, the FST was performed (see Section 4.4.3), and then the animals were divided into two groups. The first one was intended for testing locomotor activity (see Section 4.4.4), and the second one was intended for the electrophysiological experiments (see Section 4.5). The design of the behavioral part of the experiment considered the requirements and sensitivity of each of the behavioral tests used. First, the memory test (TOMT) was performed on mice that had not previously undergone any behavioral tests, which, by providing a stress factor, could negatively affect the effectiveness of learning. Next, a moderately invasive NCT was performed, and the next day, the FST, which is the most stressful in itself, was performed. The control animals were tested with the same schedule as the CUMS animals.

### 4.4. Behavioral Studies

#### 4.4.1. Temporal Order Memory Task (TOMT)

This test was conducted according to Barker et al. [38] with some modifications. In brief, the habituation phase, sample tests, and test trials took place in a dark room in a black wooden square open field (50 × 50 × 50 cm) illuminated by a 25 W bulb. The floor of the arena was covered with sawdust. The habituation trial was carried out for 10 min for 2 days, during which each mouse was placed individually in the apparatus in the absence of objects and allowed to explore the environment. Twenty-four hours later, the TOMT task comprising two training phases (TP1 and TP2) and one test trial was performed. TP1 and TP2 were performed with an interval of 2 h. In each training phase, the mice were allowed to explore two copies of an identical object for 4 min. In TP1, two O1 objects were used, and in TP2, two O2 objects were used. Two hours after TP2, the test trial lasting 3 min was performed. During this phase, a third copy of O1 and a third copy of O2 were used. If temporal order memory is intact, the subjects spend more time exploring O1 than O2. Time (T) spent exploring (i.e., sniffing or touching) objects O1 and O2 was measured by a trained observer, followed by calculation of the discrimination index [(TO1 ′ TO2)/(TO1 + TO2)].

#### 4.4.2. New Cage Test (NCT)

The NCT, performed to assess the exploration of a new environment, was carried out according to Strekalova et al. [14] with minor modifications. Mice were introduced into a standard plastic cage filled with fresh sawdust. The number of exploratory rearings was counted under red light during a 3 min period by visual observation.

#### 4.4.3. Forced Swim Test (FST)

The forced swim test was performed according to the protocol previously used in our laboratory [8]. Mice were placed individually into glass cylinders (height: 25 cm, diameter: 15 cm) containing water at 23 °C. The water column was deep enough so that the mice could not support themselves by placing their paws on the base of the cylinder. The animals were left in the cylinder for 6 min. The duration of immobility was measured during the last 4 min. The mouse was judged to be immobile when it floated passively.

#### 4.4.4. Locomotor Activity Test

The locomotor activity of the mice was measured with a 12-station photobeam activity system (Opto-M3 Activity Meter, Columbus Instruments, Columbus, OH, USA). The animals were individually placed in Plexiglas locomotor activity chambers (40 × 20 × 15 cm) and immediately thereafter, the total distance traveled during a 60 min experimental session was measured and stored every 5 min.

### 4.5. Electrophysiology

#### 4.5.1. Slice Preparation

The animals were anesthetized using isoflurane (Aerrane, Baxter, Deerfield, IL, USA). Under anesthesia conditions, the brains were rapidly removed from the skulls and prepared in NMDG-based, cold artificial cerebrospinal fluid according to the procedure described by Ting et al. [40]. Cortical slices (380 µm thick) were cut in the coronal plane using a Leica VT 1000s vibrating microtome. The slices were incubated in carbogen bubbled ACSF containing (in mM) NaCl (132), NaHCO_3_ (26), CaCl_2_ (2.5), D-glucose (10), KCl (5), MgSO_4_ (1.3), and KH_2_PO_4_ (1.25) at 32 ± 0.5 °C, then transferred to the recording chamber (interface type) and superfused (2.5 mL/min) with ACSF containing (in mM) NaCl (132), NaHCO_3_ (26), CaCl_2_ (2.5), D-glucose (10), KCl (2), MgSO_4_ (1.3), and KH_2_PO_4_ (1.25).

#### 4.5.2. Field Potential (FP) Recording, Paired-Pulse Stimulation and LTP Induction

A stimulating electrode (concentric, Pt-Ir; FHC, USA) was placed in cortical layer V. Basic stimulation (0.016 Hz frequency, duration of 0.2 ms) was applied using a constant-current stimulus isolation unit (WPI). Field potential (FP) recordings were performed using ACSF filled glass micropipettes (1–2 MΩ). Glass electrodes were placed in cortical layer II/III. The recorded responses were amplified (EXT 10-2F amplifier, NPI), then filtered (1 Hz-1 kHz), A/D converted (10 kHz sampling rate), and collected on a commercial personal computer with Micro1401 interface and Signal 4 software (CED). An input‒output (stimulus–response) curve was made for every single slice. To obtain the curve, the stimulation intensity was increased stepwise from 0 to 100 µA with 5 µA steps. One response was recorded at each stimulation intensity. Then, the stimulation intensity was adjusted to evoke a response of 30% of the maximum amplitude.

Paired-pulse stimulation, a model of short-term synaptic plasticity, was induced with two stimuli of equal intensity (approximately 30% of maximum) at a time interval of 50 ms. For the paired-pulse ratio test (PPR), two stimuli with the same intensity and duration (0.1 ms) were applied at intervals of 50 ms. PPF was expressed as the ratio of the initial amplitude of the second to the first evoked response (FP2/FP1) using the averages of three consecutive responses.

For LTP induction, theta burst stimulation (TBS) was used. TBS was composed of 10 stimuli trains at 5 Hz, repeated 5 times with 15 s gaps. The single train was composed of five 100 Hz pulses. The single pulse duration was increased to from 0.2 to 0.3 ms.

For each slice, the stimulus–response data were fit with the Boltzmann equation: Vi = Vmax/(1 + exp((u − uh)/−S), where Vmax is the maximum FP amplitude; u is the stimulation intensity; uh is the stimulation intensity evoking FP of half-maximum amplitude; and S is the factor proportional to the slope of the curve.

### 4.6. Data Analysis

All the results obtained were expressed as the mean ± standard error of the mean (SEM). GraphPad Prism 7.00 (GraphPad Software, San Diego, CA, USA) was used for the analysis of behavioral data. The effects obtained in the TOMT, NCT, and FST were analyzed using two-way ANOVA. Locomotor activity data were evaluated by repeated-measures ANOVA. Statistical analysis of the electrophysiological data was carried out using one-way analysis of variance (ANOVA) followed by Tukey’s post hoc test using GraphPad Prism 4.00 (GraphPad Software, San Diego, CA, USA). The results were considered to be significant if the *p*-values were below 0.05.

## 5. Conclusions

We conclude that the combined administration of (*R*)-ketamine and the mGlu_2/3_ receptor antagonist LY341495 (mixRL) may improve the memory dysfunction induced by depression via mechanisms involving enhancement of LTP induction and modulation of the excitatory synaptic transmission in the PFC in the CUMS model of depression. Importantly, this study not only provides new insight into the mechanism of action of the new potential RAAD but also indicates its additional beneficial effects related to the reversal of stress-induced PFC-dependent memory deficits, which are critical features of depression, and confirms its safety as a drug.

## Figures and Tables

**Table 1:** Effects of CUSM/mixRL treatments on parameters characterizing stimulus–response curves of field potentials, calculated using Boltzmann fits.

	V_max_ (mV)	u_h_ (µA)	S	N
veh	1.18 ± 0.04	27.26 ± 1.7	11.39 ± 0.9	24
CUMS	1.47 ± 0.07 ***^,#^	24.59 ± 2.33	9.82 ± 1.6	17
MixRL	1.24 ± 0.06	27.59 ± 1.33	11.65 ± 1.43	20

Data are presented as the means ± SEM. Threshold, stimulus intensity evoking FP of approx. 0.1 mV in amplitude; Vmax—maximum FP amplitude; uh—half maximum stimulation; S—factor proportional to the slope of the curve; N—number of slices. *** *p* < 0.001 veh vs. CUMS, # *p* < 0.05 mixRL vs. CUMS.

## Data Availability

All relevant data are presented in the manuscript; raw data are available upon request from the corresponding author.
